# Experimental Analysis of Commercial Optical Methods for Foot Measurement

**DOI:** 10.3390/s22145438

**Published:** 2022-07-21

**Authors:** Matthias C. Jäger, Jörg Eberhardt, Douglas W. Cunningham

**Affiliations:** 1 Institute for Photonic Systems, Ravensburg-Weingarten University, Doggenriedstraße, 88250 Weingarten, Germany; joerg.eberhardt@rwu.de; 2 Department for Graphical Systems, Brandenburg University of Technology, Konrad-Wachsmann-Allee 5, 03046 Cottbus, Germany; douglas.cunningham@b-tu.de

**Keywords:** foot measurement, time of flight, structured light, image processing, shape completion

## Abstract

Due to the increasing trend of online shopping, shoes are more and more often bought without being tried on. This leads to a strong increase in returns, which results in a high financial as well as ecological burden. To prevent this, feet can be measured either in the store or at home by various systems to determine the exact dimensions of the foot and derive an optimal shoe size. In this paper, we want to present an overview of the methods currently available on the market for the measurement of feet. The most important commercial systems are classified according to the underlying basic technology. Subsequently, the most promising methods were implemented and tested. The results of the different methods were finally compared to find out the strengths and weaknesses of each technology. After determining the measurement accuracy of the length and width for each measurement method and also comparing the general shape of the 3D reconstruction with the GT, it can be said that the measurement using a ToF sensor is currently the most robust, the easiest and, among other methods, the most accurate method.

## 1. Introduction

Increasingly, people tend to purchase clothes and shoes online. One consequence of this is that items cannot be tried on to see if they fit before they are purchased. This is particularly problematic when it comes to shopping for shoes. As a result, customers often purchase several pairs of shoes in different sizes, let the shoes be shipped and then return those pairs that do not fit. This leads to a high financial as well as environmental cost. For example, in Germany about 286 million [1] articles are returned. Of these, 18% [2] are shoes, which are usually returned because they do not fit. The average loss per return is about EUR 15.18 [1]. Furthermore, approximately 849g CO${}_{2}$ [3] is emitted per return. The massive and unnecessary total annual financial loss (about 4.34 EUR billion) and annual environmental cost (about 242,814 tons of CO${}_{2}$) cannot be overstated. One way to avoid incurring these costs would be to have a simple system to precisely measure feet from anywhere, determine the appropriate shoe size and thus reduce the number of returns. Currently, there are a large number of different systems for measuring feet and determining their size. While traditional systems use custom-made measuring tools, the majority of modern systems performed this task with the help of computer vision algorithms and standard hardware. Most of these approaches are based either on photogrammetry from two dimensional (2D) images, on measurements out of three-dimensional (3D) point clouds or on image processing using 2D RGB images. After an expert interview with the company Corpus.e, it turned out that most approaches to foot measurement at home are too complicated and costly. For this reason, we want to develop a new approach, which is suitable for untrained people. As a basis for this new method, this work will test the most promising methods for measurement, both to obtain a reference for the new application and to find a suitable optical measurement method on which to base the application.

Two-dimensional camera approaches [4,5] use a 2D image to measure the size of a foot. To be able to do this, either a reference object with known size must be in the image, or the camera position relative to the object as well as the intrinsic parameters of the camera must be known. For measurement approaches that are feasible for private persons with a smartphone, the variant with the reference is usually used. Classic reference objects here are, for example, coins or credit cards. Professional measuring systems, on the other hand, often use a calibrated, permanently installed camera whose position and intrinsic parameters are known in addition to a reference. In both variants, the size of the measured object can be measured by means of classic image processing techniques using the number of pixels that the measured object occupies in the image.

Approaches based on photogrammetry [6,7,8,9,10,11,12,13,14,15] in most cases use 2D images as input to generate a 3D model. The big advantage of photogrammetry is that the input data can be generated easily and cheaply without the need for special hardware. Furthermore, with photogrammetry it is possible to reconstruct the scanned object including color and texture. In addition, photogrammetry can scan objects of any size and has no limitations compared to other scanning technologies. However, the technology also has weaknesses, such as the fact that the quality of the reconstructions depends strongly on the texture and surface condition of the object. For example, smooth, shinny, hairy or monochrome objects are difficult to digitize [16]. Furthermore, uneven illumination in the images can lead to problems.

Another often used method for digitizing and measuring feet is 3D cameras [17,18,19,20,21,22,23,24]. There are different technologies, such as passive stereo, active stereo (e.g., structured light) and time of flight (ToF). Each of the technologies has strengths and weaknesses that can influence the result of the measurement. Passive stereo is the oldest and probably the most researched technology. RGB or gray-level images are usually used as input. Only ambient light is used to expose the image. This is also one of the biggest weaknesses of the technology because recordings in dark or dimly lit environments are not possible. To reconstruct depth information, one needs to establish a correspondence between each feature in one image with the matching feature in the other input images and then use these matches to triangulate the distance to that real-world point that gave rise to the image feature(s). Active stereo cameras are also based on triangulation, but unlike passive stereo, they often use infrared light projected with a specific pattern to illuminate the scene. However, since infrared light is also contained in sunlight, this technology is very susceptible to ambient light interference. Time of flight cameras mostly use modulated near infrared light (NIR) to illuminate the scene. The distance to the object is calculated by measuring the time the emitted signal needs to reflect back from the measured object. ToF is relatively robust to environmental effects compared to the other technologies, but it has other weaknesses such as flying pixels.

In this paper, we empirically compare the relative advantages and disadvantages of these techniques with regard to their ability to accurately measure the length and width of a foot. The different technologies are each represented by a commercial system that was used to perform the experiments. The ground truth for the examinations was generated with the 3D scanner Artec Eva [25].

## 2. Materials and Methods

In this section, we describe the framework and the methods that were used to perform the experiments.

### 2.1. Structured Light

#### 2.1.1. Operating Principle

**Figure 1:**
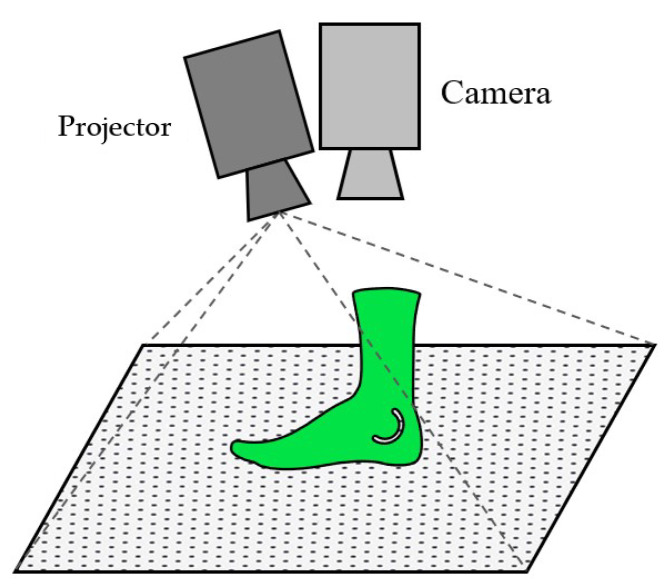
Operating principle of SL cameras with pattern projection.

Active Stereo Cameras, also known as Structured Light (SL) Cameras, use one of two techniques. Some project a modulated pattern, usually infrared light, onto a surface and calculate the distortion of the projected pattern. The others use two cameras and triangulation using corresponding or matching features in the two images. SL cameras are mostly used indoors because of interference with the infrared (IR) sunlight, which affects the projected pattern or the light used for illumination. Furthermore, in comparison to ToF cameras, SL cameras have a higher accuracy in short ranges but also a lower measurable distance. An example for state-of-the-art SL cameras is the Intel RealSense series or the Microsoft Kinect V1 [26]. Figure 1 shows the pattern, which is projected to a curved object. The pattern distorts, and the camera measures the deviation from the original pattern.

#### 2.1.2. Ground Truth—Artec Eva

To compare the accuracy of the different systems, the ground truth (GT) of the foot model was created using the Artec Eva structured light scanner. Due to its 3D point accuracy of 0.1 mm [25], this model serves as an excellent reference for the other technologies. The scanner can digitize objects from a size of 10 cm including their texture. It has a 3D build rate of 16 fps and works without targets. Due to the fact that this scanner uses multiple technologies (SL and photogrammetry), it was not considered for the actual tests, which were to look at only one method. The scanner was used with the Artec Studio 16 Professional software on a PC with a GeForce 2080 RTX graphics card. To get as close as possible to a real foot measurement scenario, the ground truth was created in a normally lit, not specially prepared room. Only the windows were darkened to prevent possible disturbing effects due to sunlight.

#### 2.1.3. Equipment and Experimental Setup—Intel RealSense D455

We selected the Intel RealSense D455 as the representative SL sensor. The camera contains two imagers used for triangulation, an IR Projector for illumination of the scene and a RGB module. The pixel size for this camera is 3 μm^2^. The maximum range is about 10m depending on calibration, scene and lightning conditions. The sensor has a FOV of H:87 (±3)/V:58 (±1)/D:95 (±3), a depth resolution of 1280 × 720 pixels with a frame rate up to 90 fps and a depth accuracy of <2% at 4 m. The resolution of the RGB sensor is 1280 × 800 pixels. The ideal distance of the camera to the measuring object is specified as 0.6 m up to 6 m.

**Figure 2:**
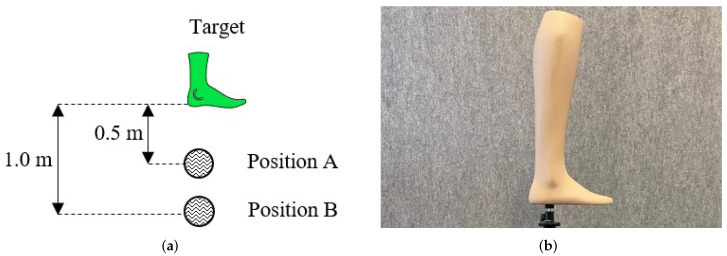
Experimental Setup for the foot reconstruction with the ToF and SL cameras: (**a**) experimental setup for the foot reconstruction; (**b**) foot model used for the reconstruction setup.

For our experiments, the camera was mounted on a tripod, positioned parallel to the camera and was placed one after the other on the different positions A-B, as shown in Figure 2a. As a target, we used a foot model, shown in Figure 2b, for measuring the error in reconstruction of an object. This test was conducted indoors. Several point clouds were recorded for each camera position. These point clouds were compared with the ground truth to determine how accurately the model could be reconstructed by the camera.

To record the point cloud, the script “rs-pointcloud.exe” from the Intel RealSense SDK 2.0 was used.

### 2.2. Time of Flight

#### 2.2.1. Operating Principle

ToF approaches [27,28] use a modulated Near Infrared (NIR) light source to illuminate the scene. The distance to the object is calculated by measuring the time the emitted signal needs to reflect back from objects in the scene. As speed of light in air is constant and known very precisely, the distance to the object can be easily calculated. Compared to other 3D camera technologies, ToF has a lot of advantages. For example, ToF is relatively insensitive to lighting conditions since the modulated signal can be subtracted from the natural ambient light. The camera consists of a lens that collects the emitted light, an image sensor that converts and processes the light into signals and an active light source that is used to illuminate the scene. Probably one of the most used ToF cameras in state-of-the-art applications is Microsoft Kinect V2. The Kinect V2 was replaced by the Kinect Azure, which was released in March 2020 [26].

##### Different Operating Approaches

Here we briefly describe the different principles of operation for ToF camera systems. There are two common methods for the illumination, namely pulsed light (PL) and continuous wave (CW).

##### Pulsed Light

Short light pulses are generated by the illumination unit by repeatedly switching it on and off. If the modulation frequency is known, the calculation of the distance for this method is straightforward because the measured phase delay corresponds directly to the time of flight [29]. As soon as the light pulse is emitted, a timer starts and measures the time it takes for the signal to be reflected and detected by the camera’s sensor [26]. The distance can be calculated as

(1) $$d=\Delta t\frac{c}{2}$$

with Δt as the time the signal takes to travel to the object and back and *c* as the speed of light which is approximately $3\times 10^{8}\frac{m}{s}$. However, the camera, which is using an infrared signal, gets affected by the ambient light, that also contains infrared light. To ensure that the calculation of the distance is not affected by this, the signal is also measured when the lighting unit is switched off. This allows us to subtract the background noise from the actual signal [26].

##### Continuous Wave Modulation

**Figure 3:**
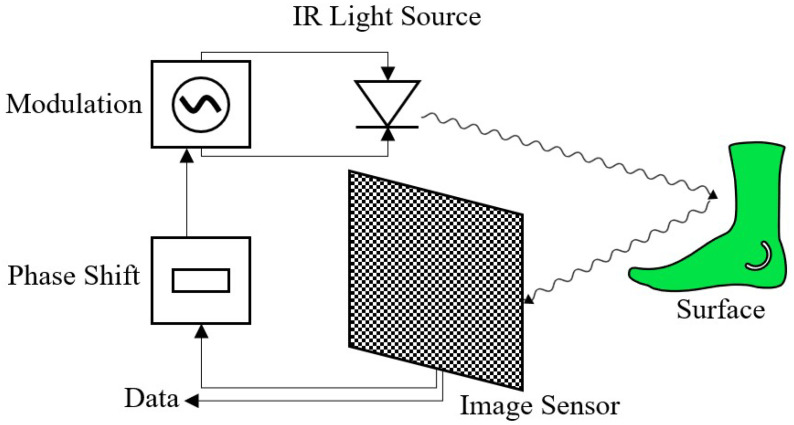
Operating principle of ToF cameras (Continuous Wave Modulation).

As can be seen in Figure 3, this method uses a modulated continuous wave of light to calculate the distance to an object. The phase shift between the emitted and received wave is measured. This phase shift is proportional to the distance of the reflecting object. Mostly either square or sinusoidal waves are used for this [30]. One advantage of CW is that it lowers the requirements for the light source in comparison to pulsed light. Thus, a higher resolution of the depth image can be realized than with pulsed light [26].

A very efficient method to demodulate the received signal is to sample the returned modulated light at four different phases (0°, 90°, 180°, 270°) [31]. For each pixel of the calculated phase image, the incoming signal is sampled and matched up with the internal reference signal. The result is a phase image of the pixel-wise sampling of the correlation function with an additional phase delay [32]. Assuming the signal g(t) is sinusoidal, discrete Fourier transform equations (also known as the 4-bucket method) can be used for the calculation of the distance [26].

From the phase ϕ and the speed of light *c*, the distance *d* to the object can be calculated as

(2) $$d=\frac{c}{4\pi f}\phi$$

#### 2.2.2. Equipment and Experimental Setup—Kinect Azure

We selected the Microsoft Kinect Azure as the representative ToF system. It implements the Amplitude Modulated Continuous Wave (AMCW) ToF principle [33,34]. For the illumination of scenes in near and wide field of view modes, two NIR laser diodes are used. Furthermore, one of the key features of the camera is the 1 Megapixel ToF imaging chip, which enables higher modulation frequencies and better depth precision. This allows a pixel size of 3.5 μm${}^{2}$.

The camera supports 2 × 2 binning modes. The use of these modes enables an extension of the z range at the expense of depth resolution. The narrow field of view modes (NFOV UNBINNED and NVOV 2X2BINNED) are made for scenes with smaller X- and Y extends but smaller Z-dimensions. In comparison, the wide field of view modes (WFOV UNBINNED and WFOV 2X2BINNED) are better suited for scenes with large X-Y extends but smaller Z-ranges [33]. The binning modes allow the Z-range to extend at the expense of resolution. To generate the point clouds, the “fastpointcloud.cpp” script included in the Azure-Kinect-Sensor-SDK was used to ensure repeatability by others. The only change to the code was to switch the sensor mode to NFOV UNBINNED and the frames per second to 15. These settings were selected for the following reasons:

- **WFOV**: This mode was used because with a wide field of view, from a short distance, more information is contained in the image. The operating range of 0.25 m to 2.21 m described in the documentation is also best suited to our planned application.
- **Unbinned**: We decided to use the Unbinned mode because it has a higher resolution.
- **15 fps**: We chose 15 fps because this mode offers a significantly higher number of measurement points in the point cloud compared to operation at 30 fps.

Then, for both the measurement of the length and the measurement of the width of the foot, 10 point clouds were recorded to determine the average error in the measurement as well as the standard deviation. The remaining experimental setup in this experiment is the same as described earlier in Section 2.1.3.

### 2.3. 2D Camera System

#### Equipment and Experimental Setup—Feetbox Evo

**Figure 4:**
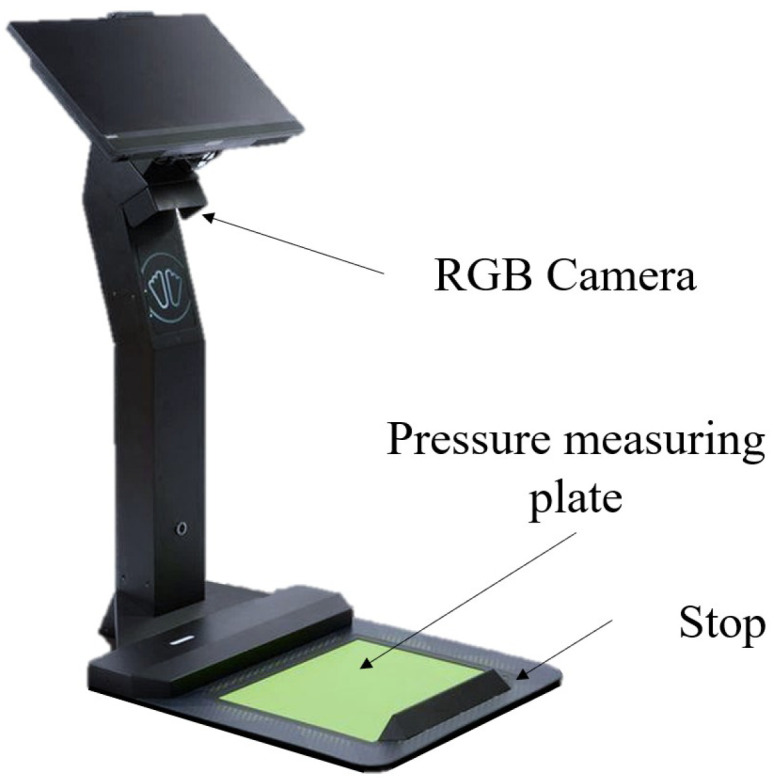
Feetbox Evo Scanner for foot size determination [35].

To investigate the accuracy of reconstructing a foot using an 2D RGB sensor, the Feetbox Evo from Corpus.e was used. Although an RGB sensor is used here, the function of the sensor to detect colors is not relevant for the process. Because color filters are used, the resolution is even lower than if only a gray scale image were used. In this case, the RGB image is only used to visualize the foot for the user. As can be seen in Figure 4, the system uses a centrally mounted camera, which is directed at the green measuring surface. To be able to determine the length and width of the feet, the feet must be placed against the stop of the measuring apparatus. An image is then taken, from which all relevant quantities can be extracted.

The camera installed in this system is the IDS UI-1251LE-C-HQ. This is a CMOS color camera with 1.92 Mpix. The resolution of the camera is 1600 × 1200 pixels.

### 2.4. Photogrammetry

#### 2.4.1. Operating Principle

Photogrammetry is a measurement approach that uses a variety of images captured by imaging sensors such as CCD, CMOS [36] to identify patterns and features in the images. The illumination of the scene can be performed with visible, as well as invisible light such as IR. The features on the photographed object are found and tracked to detect deformations in the geometry of the object. This information is used to align the images in 3-dimensional space. With the help of trigonometric calculation, the topology of the 3D object can thus be calculated.

#### 2.4.2. Equipment and Experimental Setup—Agisoft Metashape

We selected the Agisoft Metashape [37] software as the representative photogrammetry system. For the acquisition of the images, an iPhone 11 Pro camera was used. A smartphone was used because the application for which this research was conducted is intended to run on a smartphone. An iPhone was used as Apple has had the largest stake in the smartphone market in recent years [38]. The smartphone has a 12 MP camera system that consists of three cameras with different lenses. These are an ultra-wide-angle lens, a wide-angle lens and a telephoto lens. The wide-angle camera with an aperture of f/1.6 was used for the test. The resolution of the resulting images is 3024 × 4032, and Smart HDR was used.

The experiment was explicitly conducted from the perspective of an untrained user to simulate the latter application. For this reason, no special emphasis was placed on perfect recording quality. The pictures were therefore taken freehand and not with a tripod or similar.

## 3. Results for Length and Width Measurements

**Figure 5:**
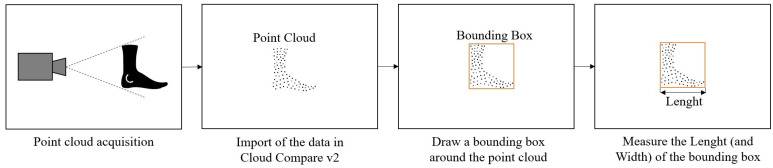
Experimental procedure for length and width measurement.

The Cloud Compare v2 program was used to evaluate the results of the different measurement systems. The point clouds were aligned, and a bounding box was created. The length and width of the foot were determined using this bounding box, as illustrated in Figure 5. The measured length was then compared to the ground truth, and an error value was determined. The results for the different systems are shown below.

To later determine an accurate shoe size from the measured values of the different systems, the error in the measurement must be smaller than 3.33 mm, which corresponds to half a shoe size in the European system [39].

### 3.1. Active Stereo

#### 3.1.1. Quantitative Analysis of Length and Width Measurement with Active Stereo

**Figure 6:**
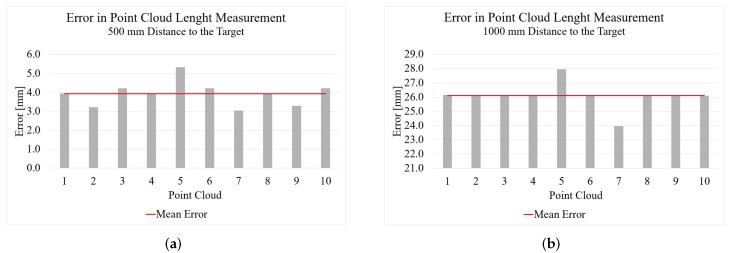

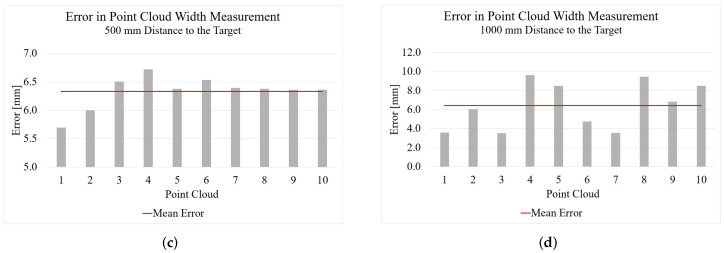
Results of the foot measurement with the Intel RealSense D455 SL camera: (**a**) length results from 500 mm; (**b**) length results from 1000 mm; (**c**) width results from 500 mm; (**d**) width results from 1000 mm.

Figure 6 shows the measurement results from Intel’s Structured Light camera. On the far left in green is the ground truth of the measured object generated with the Artec Eva. In the middle is the point cloud generated with the Intel RealSense D455 from a distance of 500 mm and on the far right the point cloud from a distance of 1000 mm. When acquiring the point clouds, the camera was positioned parallel to the measurement object and at the same height level as the object. It can be seen that from a distance of 500 mm there is already a mean error to the actual length of the foot of 3.9 mm. From 1000 mm, this result for length measurement deteriorates further with an error of 26.1 mm. When measuring the foot width, a mean error of 6.3 mm occurs from a distance of 500 mm to the foot. Surprisingly, this error stays almost the same (6.4 mm) from a distance of 1000 mm. From a distance of 1000 mm, however, the measured values are no longer as constant as from 500 mm.

#### 3.1.2. Qualitative Analysis of Length and Width Measurement with Active Stereo

**Figure 7:**
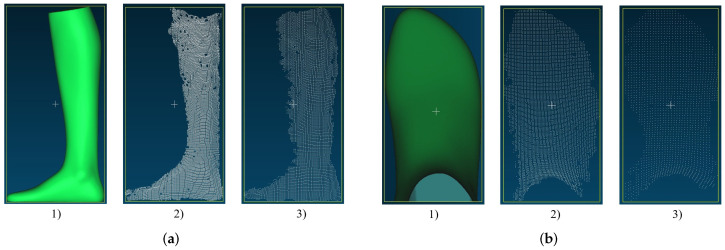
Example of point clouds recorded with Intel RealSense D455 SL camera: (**a**) point clouds of the foot model generated for length measurement: (1) GT, (2) length measurement (500 mm), (3) length measurement (1000 mm); (**b**) point clouds of the foot model generated for width measurement: (1) GT, (2) width measurement (500 mm), (3) width measurement (1000 mm).

Figure 7a shows the results of the foot reconstruction with the Intel RealSense D455. The reconstruction accuracy of the camera changes significantly with increasing distance to the object. From a distance of 500 mm, the point cloud is already full of holes, but it still resembles the GT. At a distance of 1000 mm, the edges of the point cloud become much more frayed. The same behavior can be observed when measuring the width.

Looking at the results of the width measurement with the Intel RealSense D455 in Figure 7b, we can also see here that the point cloud from 500 mm loses density at the edges. However, the rough foot shape can still be seen. When measured from 1000 mm, the shape of the point cloud is very different from the GT. However, as mentioned before, the error in the width measurement is surprisingly smaller than in the measurement from 500 mm.

### 3.2. Time of Flight

#### 3.2.1. Quantitative Analysis of Length and Width Measurement with Time of Flight

**Figure 8:**
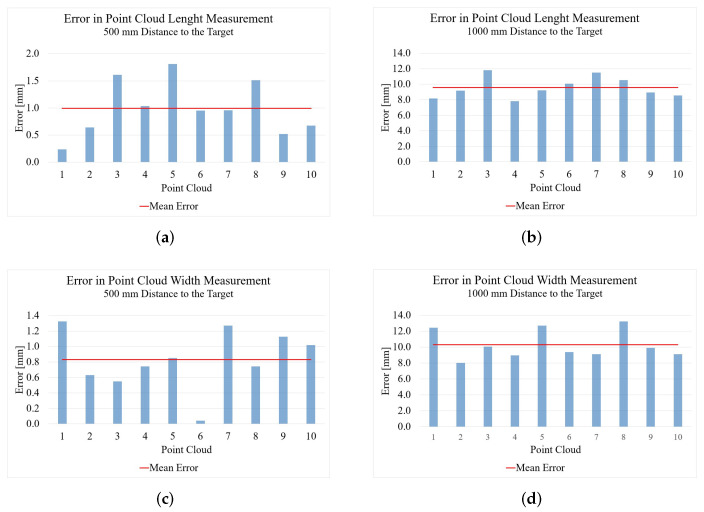
Results of the foot measurement with the Microsoft Kinect Azure ToF camera: (**a**) length results from 500 mm; (**b**) length results from 1000 mm; (**c**) width results from 500 mm; (**d**) width results from 1000 mm.

Figure 8 shows the measurement results of the point clouds generated by the Microsoft Kinect Azure. The reconstruction has an error of 0.5 mm from a 500 mm distance to the target.

From a distance of 1000 mm, the result of the length measurement gets worse, with an error of 7.6 mm. With the width measurement, the result from 500 mm, with an error of 1.4 mm, is not quite as good as with the length measurement but still nearly perfect. With the greater distance of 1000 mm, the result here, with an error of 7.2 mm, is significantly worse.

#### 3.2.2. Qualitative Analysis of Length and Width Measurement with Time of Flight

**Figure 9:**
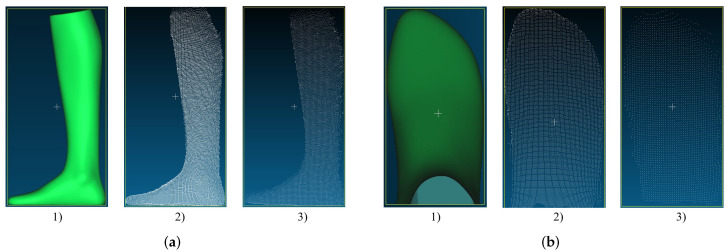
Example of point clouds recorded with the Microsoft Kinect Azure ToF camera: (**a**) point clouds of the foot model generated for length measurement: (1) GT; (2) length measurement (500 mm); (3) length measurement (1000 mm); (**b**) point clouds of the foot model generated for width measurement: (1) GT; (2) width measurement (500 mm); (3) width measurement (1000 mm).

Figure 9a shows the point clouds for length measurement generated by the Microsoft Kinect Azure. The uniformity of the point clouds is much higher than was the case for the Intel RealSense D455. There are no holes, and the edges of the point clouds are clearly defined and not frayed. The overall shape of the point cloud is very good and very similar to that of the GT, especially from a distance of 500 mm. From a distance of 1000 mm, the point cloud is getting more frayed, almost like with the RealSense D455. However, the shape of the foot is still clearly recognizable.

The point clouds of the Kinect Azure, which were created for the measurement of the foot width, can be seen in Figure 9b. Again, similar to the length measurement, the density of the point cloud decreases from a distance of 500 mm to 1000 mm. The overall shape is still recognizable, but the edges look much more frayed from the 1000 mm distance, especially in the toe area. In contrast to the point cloud from 500 mm, which is very similar to the GT except for a few flying pixels on the left side, you can see that especially the edges of the point cloud from 1000 mm could not be reconstructed very well. This observation is also reflected in the measurement results.

### 3.3. 2D Camera System

#### 3.3.1. Quantitative Analysis of Length and Width Measurement with a 2D Camera System

Table 1 shows the results of the length and width measurement with the Feetbox Evo, which was performed on the basis of a single RGB image. Since the result is rounded, the measurement error cannot be determined as precisely as in the previous measurements. The measured error compared to the GT is 0.2 mm for the length measurement and 0.5 mm for the width measurement. In both cases, the error is extremely small compared to the other measurement methods, which indicates the effectiveness of this measurement method.

#### 3.3.2. Qualitative Analysis of Length and Width Measurement with a 2D Camera System

An optical comparison of the 3D data cannot be performed in this case since no 3D data is generated with this approach. The length and width are determined with the help of a calibrated and permanently installed camera, as well as with the help of the stop (see Figure 4).

In addition to the optical measurement of the foot, the scanner also generates a pressure profile of the foot showing the different load zones of the sole of the foot. This profile is generated by using a pressure measurement plate built into the scanner. However, this data was not used in this test series.

### 3.4. Photogrammetry

**Figure 10:**
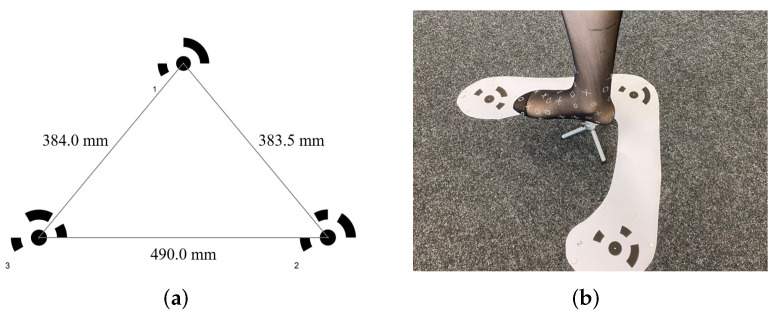
Experimental setup for photogrammetry: (**a**) target arrangement; (**b**) example image used for photogrammetry.

For the reconstruction of the foot model, 41 images of the foot were acquired from different positions. Since the procedure did not work with the foot model itself, which is too smooth and has too little texture, a very thin sock with a drawn pattern was placed on the foot as seen in Figure 10b, which should not have much influence on the shape of the foot. To properly scale the foot in Agisoft Metashape, the arrangement of targets in Figure 10 was used. The distance between the targets was measured to use this information as a size reference in Metashape. The targets were cropped to remove as much white area as possible because the algorithm has difficulty or even no possibility to use areas without texture.

In contrast to the other experiments, this one is difficult for a private person to perform since the experiment here is very time-consuming as well as computationally expensive. However, to evaluate the accuracy of the reconstruction using professional photogrammetry software, this experimental setup was chosen. Although there are already simpler methods of performing photogrammetry using an app directly on the smartphone, a conscious decision was made not to do so here, as the result of the photogrammetry should be checked under the best possible conditions.

#### 3.4.1. Quantitative Analysis of Length and Width Measurement with Photogrammetry

**Figure 11:**
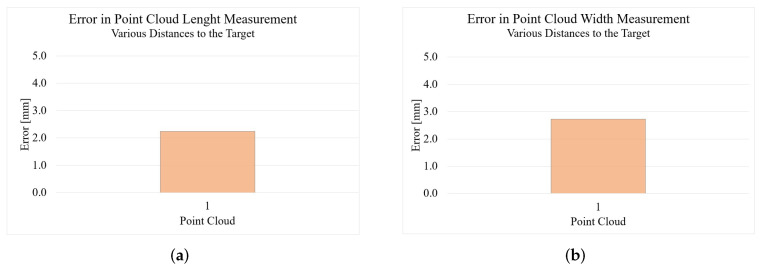
Results of the foot measurement with Agisoft Metashape: (**a**) result length; (**b**) result width.

Figure 11 shows the results of the measurements of the reconstruction using photogrammetry. Due to the high complexity of the experiment, only one point cloud was created here. With an error of 2.24 mm in the length measurement and 2.73 mm in the width measurement, the results here are very good. However, it must be noted that the measured values can only be as good as the measurement of the reference variables. If these references are given incorrectly, the result will also be unsatisfactory.

#### 3.4.2. Qualitative Analysis of Length and Width Measurement with Photogrammetry

**Figure 12:**
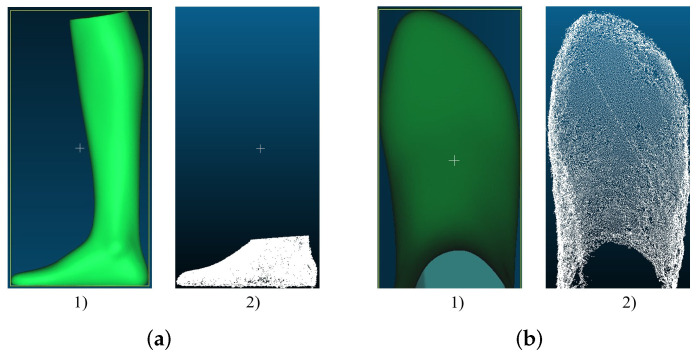
Results of the reconstructed foot with Agisoft Metashape Photogrammetry Software: (**a**) point cloud of the foot model generated for length measurement: (1) GT; (2) Agisoft Metashape; (**b**) point cloud of the foot model generated for width measurement: (1) GT; (2) Agisoft Metashape.

Figure 12a,b show the reconstructions in comparison to GT. It can be seen that the general shape is in good agreement with the GT. Only the bottom side of the foot cannot be reconstructed that well, since no pictures of the bottom side can be taken. However, the results are still so good that additional images of the bottom of the foot were not necessary.

## 4. Results for the Overall Reconstruction Shape

**Figure 13:**
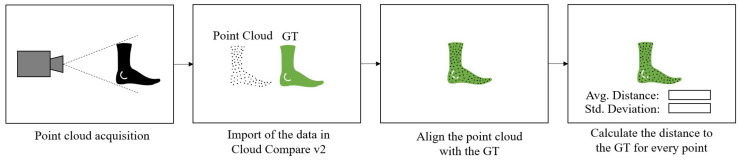
Experimental procedure for the overall shape reconstruction.

In this section, the general shape of the reconstruction of the foot model is directly compared to the GT. For this purpose, the generated point cloud was overlaid on the GT in the Cloud Compare v2 software. Cloud Compare v2 was used to ensure comparability with other studies such as [40]. To best fit the point cloud over the GT, it was roughly aligned for the initial stage. Afterwards, an algorithm integrated in Cloud Compare was used for fine-tuning. This minimizes the root mean square so that all points lie optimally on the GT. Afterwards, the average distance of all points to the GT is calculated. The workflow described is shown in Figure 13. To evaluate the results, the maximum distances of the point cloud to the GT were considered. Therefore, the error values describe the maximum distances of the reconstructed geometry to the ground truth. The aim here is to determine whether there are large outliers in the measurement data that negatively affect the reconstruction of the shape.

Since the result of the last experiment was that the quality and therefore the accuracy of the point cloud was highest for all systems from a distance of 500 mm, only these point clouds were used for this experiment.

### 4.1. Active Stereo

**Figure 14:**
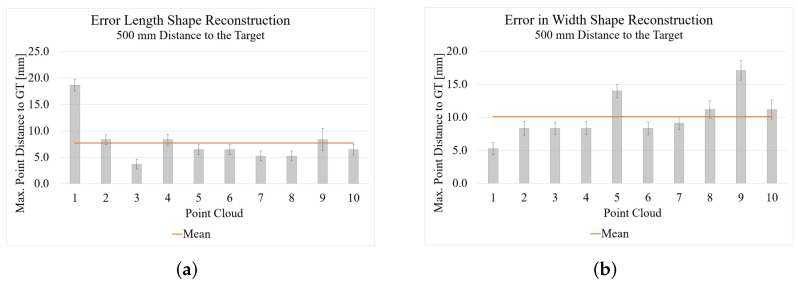
Results of the shape reconstruction of length and width with the Intel RealSense D455: (**a**) length reconstruction; (**b**) width reconstruction.

Figure 14 shows the result of reconstructing the shape with the Intel RealSense D455. The average of the maximum distance of the points to the GT is 7.75 mm for the length measurement and 10.14 mm for the width measurement are shown. The average standard deviation is 1.10 for the length measurement and 1.11 for the width measurement. It is interesting that matching with the GT gives such a good result, since the results of the length and width measurement are behind in comparison with the Kinect Azure. The reason for this is explained in the results for the Kinect Azure in Section 4.2.

**Figure 15:**
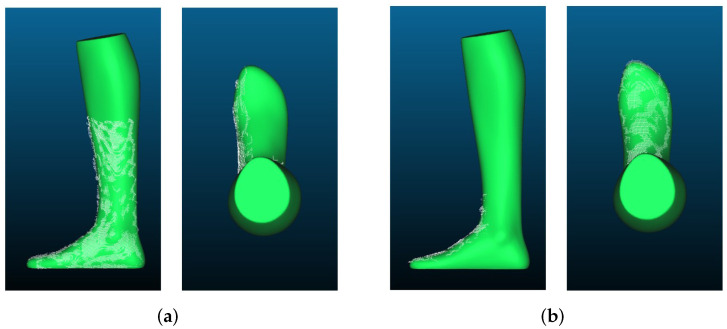
Point clouds recorded with Intel RealSense D455 placed over the GT: (**a**) length reconstruction; (**b**) width reconstruction.

In Figure 15, you can see the point clouds compared to the GT. It can be seen that the point clouds look very similar to the GT and that the overall shape of the foot has been well reconstructed except for some outliers.

### 4.2. Time of Flight

**Figure 16:**
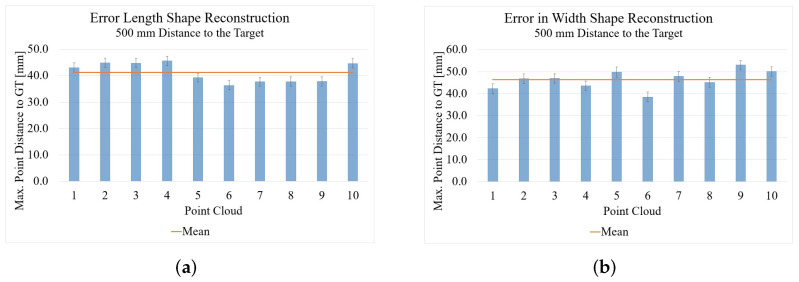
Results of the shape reconstruction of length and width with the Microsoft Kinect Azure: (**a**) length reconstruction; (**b**) width reconstruction.

The results of the point clouds recorded with the Kinect Azure compared to the GT can be seen in Figure 16. The average maximum point distances of the measurements with the Kinect Azure for the length measurement are 41.17 mm and for the width measurement 46.40 mm. The standard deviation for the point cloud of the length measurement is 1.74 and that of the width measurement is 2.21. It is first of all surprising that the Kinect result is worse than that of the Intel RealSense since the Kinect shows better results in the length and width measurement as well as in the optical comparison of the point clouds (Figure 7 and Figure 9). This is because the Kinect with its ToF technology has errors such as flying pixels. These flying pixels cause outliers especially at the edges of the object, which result in the relatively large error values seen in Figure 16. Since the RealSense D455 SL camera does not have these interfering pixels, the results are better for now even though they look worse opticall. However, the disturbing pixels could still be filtered and removed so that the result would be significantly improved.

**Figure 17:**
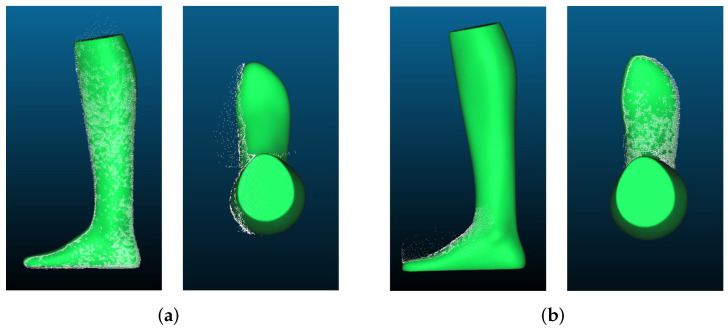
Point clouds recorded with Kinect Azure placed over the GT: (**a**) length reconstruction; (**b**) width reconstruction.

Comparing the point clouds visually with the GT in Figure 17, it can be observed that the shape has been reconstructed very well, and for a distance of 500 mm, it is almost identical to the GT. The only downfall is the flying pixels on the edges of the foot, as mentioned above.

**Figure 18:**
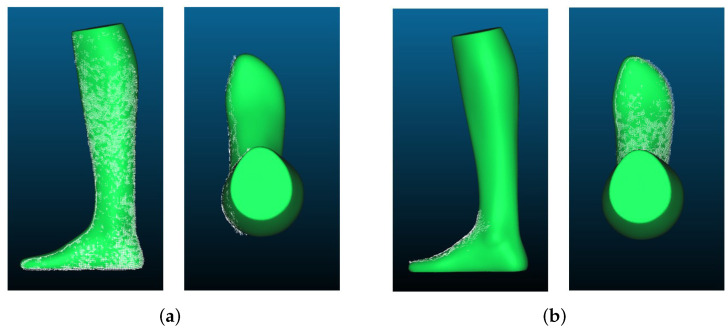
Filtered point clouds recorded with Kinect Azure placed over the GT: (**a**) filtered length reconstruction; (**b**) filtered width reconstruction.

To test how good the Kinect results would be without the flying pixels, the point clouds in Cloud Compare were filtered using the SOR (Statistical Outlier Removal) filter. The filtered point clouds are shown in Figure 18. It can be clearly seen that the point cloud has much fewer interfering pixels; thus it fits the GT much better. Moreover, the maximum distance decreases significantly, e.g., from 43.05 mm to 7.52 mm, for the first point cloud of the length measurement. Likewise, when measuring the maximum distance to the GT of the first point cloud of the width measurement, the result improves from 42.27 mm to 5.28 mm.

This shows that the relatively weak values of the Kinect are mainly due to the errors caused by the flying pixels at the edges of the object. After filtering, the Kinect result is at a very promising level both visually and statistically.

### 4.3. 2D Camera System

Since this measuring system only works with 2D data and therefore no 3D data is available, the general shape cannot be compared with the GT here.

### 4.4. Photogrammetry

**Figure 19:**
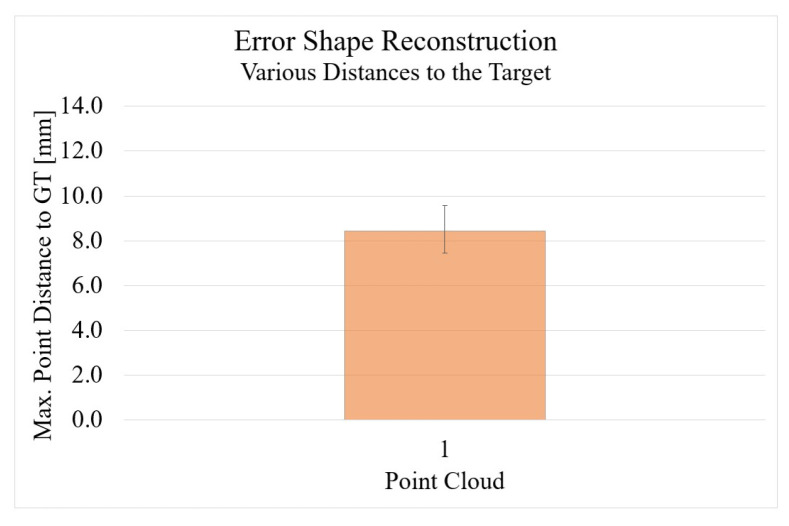
Error in overall 3D reconstruction for the photogrammetry procedure with Agisoft Metashape.

The results from the comparison of the shape between the photogrammetry reconstruction and the GT can be seen in Figure 19. With a maximum error of 8.45 mm, the photogrammetry measurement method is in the middle of the examined methods. The standard deviation is 1.13.

**Figure 20:**
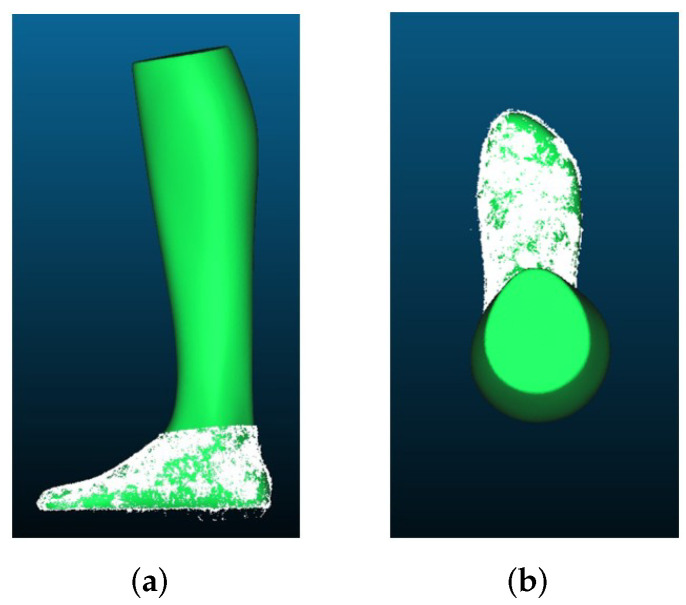
Point clouds recorded with an iPhone 11 Pro and processed with Agisoft Metashape: (**a**) length reconstruction; (**b**) width reconstruction.

It can also be seen in Figure 20 that the point cloud and the GT match very well. Only at the bottom of the foot is the reconstruction slightly worse. This is due to the fact that it was not possible to move the foot during the experiment and photograph it from below. Therefore, outliers occur here which negatively influence the result. However, as described in advance, this does not have a particularly large impact on the measurements we perform.

## 5. Conclusions

Table 2 and Table 3 show the quantitative results of the two experiments in a compact form. It is again clearly evident that the ToF method gives excellent results. The only method that provides even better results for length and width measurements is the 2D camera system. Here, however, the disadvantage is that no three-dimensional data is obtained. In third place is photogrammetry, which gives good results but is extremely complex, and the results are strongly based on the reference object in the image. The method with the SL camera performed the worst since it does not manage well with increasing distance to the measurement object, as well as with ambient lighting.

## 6. Discussion

The purpose of this study was to empirically determine which optical measurement method is most effective and accurate for measuring feet. We examined the most important optical measurement methods. Specifically, we examined Structured Light cameras, Time of Flight cameras, a single 2D camera, and photogrammetry from many 2D images. For each types of measurement method, a representative system was chosen with which the experiments were carried out. As a basis for the future determination of an appropriate shoe size, the foot length and the foot width were examined in the first experiments. Since the photogrammetry method studied is based only on 2D images without a reference from which the size could be extracted, a second experiment was performed comparing the overall shape of the reconstruction with the GT. In addition, to later match the foot with a shoe, it may be important to use a high quality reconstruction of the foot rather than relying on just a few measurements. An illustration of the advantages and disadvantages of the systems can be seen in Table 4.

The tests with the Structured Light camera brought the realization that this technology is extremely dependent on the ambient conditions and is therefore unsuitable for a mobile system for private persons, since they will rarely or never be in optimally shielded environments. Moreover, the significant deterioration of the results with increasing distance to the measurement object speak against the use of this technology. The comparison of the general shape also highlights how much the quality of the image suffers with increasing distance. However, one advantage of the camera is that it is relatively cheap and small. Moreover, Structured Light cameras are often built into smartphone front cameras for Face ID (using the iPhone as an example), which would be an advantage for developing an application for private individuals.

The results from Microsoft’s ToF camera, the Kinect Azure, showed excellent results in measuring the length and width of the foot. The reconstruction of the general foot shape is also very good. However, the camera suffers from classic flaws of the ToF technology, such as the flying pixels. Nevertheless, these can be removed by filtering outliers. Because of the way it works, the technology is also much more robust against ambient light. Thus, bright and dark scenes are no problem. Similar to the SL camera, ToF sensors are also increasingly being built into smartphones, which makes this technology easily accessible to private persons.

The measurement using an RGB image and image processing, which was carried out with the Feetbox Evo, shows very good measurement values for the length and width of the foot. However, since no 3D data is generated, only the length and width of the foot can be used for shoe sizing. Other possibly important values, such as the instep height, cannot be included. Since this method only requires an RGB image and a reference as input, the acquisition of data is extremely easy and can be performed by untrained persons.

The last method of measurement studied is the reconstruction of the foot using photogrammetry. The method provides good results, but the entire process is extremely time-consuming and complex compared to the other methods. A big advantage of this method is that you are very flexible in the selection of your input data since the algorithm can work with a variety of data formats. Moreover, only a cheap RGB camera is needed to get a result. However, the fact that a relatively large amount of data is required makes the method much less convenient.

In summary, it can be said that all methods have advantages and disadvantages. The most promising for the development of a foot measurement device for private individuals is probably the use of a ToF camera. Two-dimensional images are easier to acquire but lack information on data such as instep height. Photogrammetry requires too much input data compared to ToF to develop a convenient method, and SL camera technology is not robust enough to work reliably in any location. For all these reasons, a measurement using ToF is the best method in our opinion. The spread of this technology is also significantly increased by the increasing integration in smartphones, as already described above.

## Figures and Tables

**Table 1:** Results of the foot measurement with the Feetbox Evo RGB camera measuring system.

Measured Data	Ground Truth	2D Camera System
Length	227.8 mm	228.0 mm
Width	81.5 mm	81.0 mm

**Table 2:** Comparison of the measurement results.

Measurement	Avg. Error SL [mm]	Avg. Error ToF [mm]	Avg. Error Single RGB [mm]	Avg. Error Photogrammetry [mm]
**Length 500 mm**	3.9	0.5	0.2	2.2
**Length 1000 mm**	26.1	7.6		
**Width 500 mm**	6.3	1.4	0.5	2.7
**Width 1000 mm**	6.4	7.2		

**Table 3:** Comparison of the shape comparison results.

Measurement	SL	ToF	Single RGB	Photogrammetry
**Max. Error (Length) [mm]**	7.75	41.17/7.52	-	8.45
**Max. Error (Width) [mm]**	11.14	46.40/5.28	-	

**Table 4:** Comparison of the advantages and disadvantages of the different methods.

	Pros	Cons
**Structured Light**	- Already built into many smartphones (good availability)	- Extremely dependent on abient light - Deterioration of the results with increasing distance
**Time of Flight**	- Excellent measurement results - Robust against ambient light - In the future available in many smartphones	- Technology related errors (e.g., flying pixel)
**2D Camera System**	- Excellent measurement results - Only one inexpensive 2D camera needed	- No 3D data - Intrinsic and extrinsic parameters needed - Known size reference needed in image
**Photogrammetry**	- Only one inexpensive 2D camera needed	- Complex and time consuming execution - Expensive software - Known size reference needed in images

## Data Availability

Not applicable.

## Abbreviations

The following abbreviations are used in this manuscript:

MDPI	Multidisciplinary Digital Publishing Institute
DOAJ	Directory of open access journals
TLA	Three letter acronym
LD	Linear dichroism
GT	Ground Truth
NIR	Near Infrared
ToF	Time of Flight
SL	Structured Light
CW	Continuous Wave
PL	Pulsed Light
AMCW	Amplitude Modulated Continuous Wave
FMCW	Frequency Modulated Continuous Wave
CMOS	Complementary Metal-Oxide Semiconductor
CCD	Charge-Coupled Device
RGB	Red Green Blue
